# The New Structure of Core Oligosaccharide Presented by *Proteus penneri* 40A and 41 Lipopolysaccharides

**DOI:** 10.3390/ijms19030676

**Published:** 2018-02-28

**Authors:** Agata Palusiak, Anna Maciejewska, Czeslaw Lugowski, Antoni Rozalski, Marta Kaszowska

**Affiliations:** 1 Laboratory of General Microbiology, Institute of Microbiology, Biotechnology and Immunology, University of Lodz, PL-90-237 Lodz, Poland; agata.palusiak@biol.uni.lodz.pl; 2 Hirszfeld Institute of Immunology and Experimental Therapy, Polish Academy of Sciences, R. Weigla 12, PL-53-114 Wroclaw, Poland; anna.maciejewska@iitd.pan.wroc.pl (A.M.); lugowski@iitd.pan.wroc.pl (C.L.); 3 Department of Biotechnology and Molecular Biology, University of Opole, PL-45-035 Opole, Poland; 4 Department of Biology of Bacteria, Institute of Microbiology, Biotechnology and Immunology, University of Lodz, 90-237 Lodz, Poland; antoni.rozalski@biol.uni.lodz.pl

**Keywords:** anti-conjugate serum, core oligosaccharide, lipopolysaccharide, NMR spectroscopy, ESI MS, *Proteus penneri*

## Abstract

The new type of core oligosaccharide in *Proteus penneri* 40A and 41 lipopolysaccharides has been investigated by ^1^H and ^13^C NMR spectroscopy, electrospray ionization mass spectrometry and chemical methods. Core oligosaccharides of both strains were chosen for structural analysis based on the reactivity of LPSs with serum against *P. penneri* 40A core oligosaccharide–diphtheria toxoid conjugate. Structural analyses revealed that *P. penneri* 40A and 41 LPSs possess an identical core oligosaccharide.

## 1. Introduction

*P. penneri* are human-opportunistic pathogens causing, in preferred conditions, several types of infections among which urinary tract and wound infections are predominant. These Gram-negative bacteria produce many virulence factors including LPS (endotoxin), which seems to be the most dangerous due to its contribution to septic shock [1]. LPS of smooth bacterial strains consists of three regions: lipid A, a core oligosaccharide (OS) and an *O*-polysaccharide (*O*-PS, *O*-antigen). Only the last two have been described for the *P. penneri* species [2,3,4]. Although the *P. penneri* core oligosaccharide is characterized by lower structural diversity than the *O*-PS part (over 26 chemotypes), it is still structurally heterogeneous (12 different structures of the outer core region and a few variants of its inner part) [2,3,4,5,6]. The LPS core region may be masked by an *O*-polysaccharide, but its exposition on bacterial cells is still accessible for specific immunoglobulins. This fact was confirmed by the detection, in polyclonal rabbit antisera against *Proteus* strains, of anti-core-specific antibodies recognizing low-molecular-mass LPS species not only of homologous but also heterologous antigens [7,8,9]. This observation encouraged the examination of different *P. penneri* LPSs, in search of antigen groups with identical or similar serological activities of their core oligosaccharides, which would complete the *Proteus* classification scheme with the data on the core region serotypes. To date, 11 groups of LPS presenting one core serotype have been classified [10]. In this paper, the results of serological studies and structural analysis are presented to show another type among *P. penneri* LPSs with a common sero- and chemotype of their core oligosaccharides.

## 2. Results

### 2.1. Serological Studies

**Figure 1:**
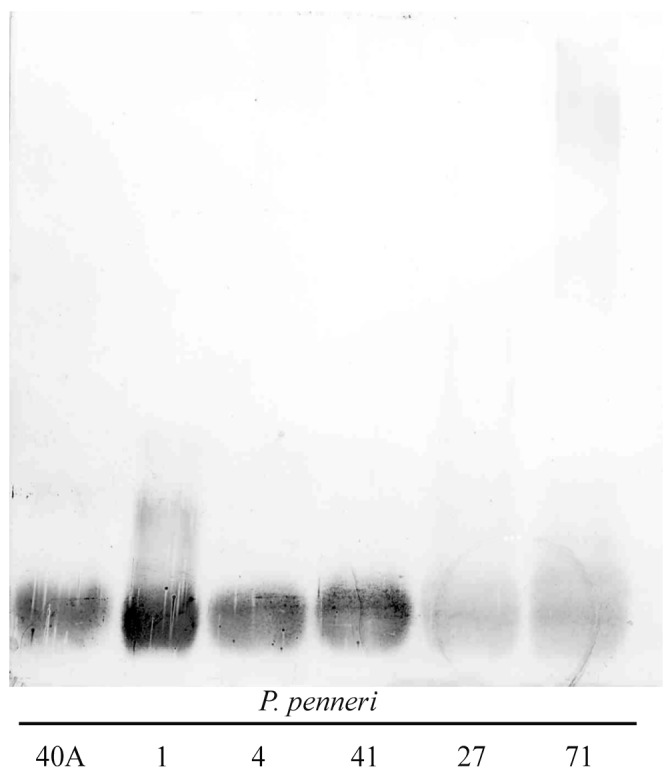
Western blot of *P. penneri* LPSs with the *P. penneri* 40A anti-conjugate serum.

The rabbit polyclonal serum against the *P. penneri* 40A core oligosaccharide–diphtheria toxoid conjugate (anti-conjugate serum) was obtained and tested by ELISA assay and immunoblotting (Western blot) with the homologous and 40 other *Proteus* spp. LPSs. The heterologous LPSs (rough *P. penneri* strains: (serotypes 4, 5, 11), R mutant of *P. mirabilis* (serotype 6) and smooth *P. penneri* strains (O8, O17, O19a,b, O31a, O31a,b, O52, O58, O59, O61, O62, O63, O64a,b,c, O64a,c,e, O65, O67–O71, O72a, O73a,b, O73a,c) representing different O serogroups and subgroups of the genus were selected as described previously [7]. In ELISA, two LPSs, *P. penneri* 40A and 41, reacted to the titer the most strongly with the tested serum (1:16,000); two LSPs, *P. penneri* 1 and 4, cross-reacted to the titer (1:8000); two other LPSs, *P. penneri* 27 and 71 showed the lowest serum reactivity titers (1:2000). Residual *Proteus* spp. LPSs were not cross-reacted with the tested serum. The cross-reactivity of the tested antiserum with *P. penneri* 1, 4, 27, 71 LPSs indicates the presence in their core oligosaccharides of similar epitope(s) common with core oligosaccharide of the homologous LPS. The strongly reacting LPSs, *P. penneri* 40A and 41, were chosen for further study. In the Western blot technique, all reactions concerned the low-molecular-mass LPS fragments consisting of the core-lipid A moieties of tested antigens (Figure 1). The tested antiserum was adsorbed a few times with an alkali-treated cross-reacting or homologous antigen and checked once more in ELISA with the same LPS preparations. The adsorption of *P. penneri* 40A anti-conjugate serum with each of the reacting LPSs completely abolished the reactions with tested LPSs. *P. penneri* 40A and 41 LPSs, which reacted strongly and similarly in all assays, have been selected for structural studies by ESI mass spectrometry and NMR spectroscopy to check the similarity of these core oligosaccharides.

### 2.2. Structural Studies

**Figure 2:**
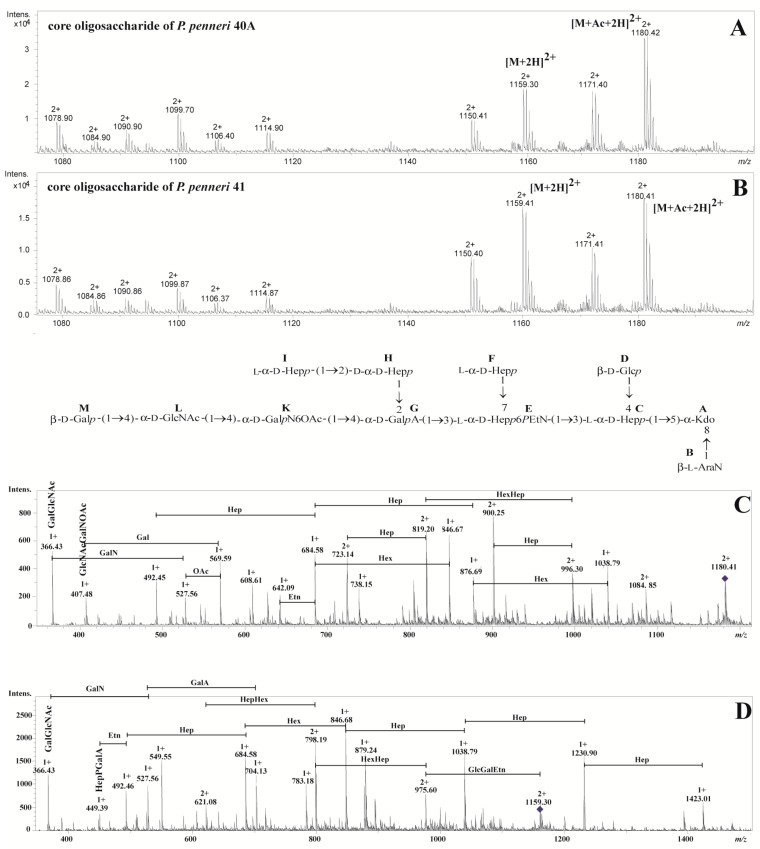
Positive ion mode ESI mass spectra of the core oligosaccharides from *P. penneri* 40A (**A**) and 41 (**B**); (**C**) Positive ion mode ESI MS/MS of the core oligosaccharide from *P. penneri* 40A represented by ions at *m*/*z* 1180.42 and (**D**) at *m*/*z* 1159.30.

The mass spectra obtained for the *P. penneri* 40A and 41 core oligosaccharides showed a high degree of similarity (Figure 2A,B). To avoid unnecessary duplication, only data concerning the *P. penneri* 40A core oligosaccharide have been presented in the text. Table 1 presents an interpretation of all ions in core oligosaccharide fractions which have been identified by ESI MS. The major fraction represented by the ions at *m*/*z* 1180.42 [M+Ac+2H]^2+^ and *m*/*z* 1171.40 [M+Ac-H_2_O+2H]^2+^ corresponded to the core oligosaccharide containing two hexoses (Glc and Gal); five heptoses (Hep); hexuronic acid (GalA); hexosamine (GalN); *N*-acetylated hexosamine (GlcNAc); 4-amino-4-deoxyarabinose (Ara4N); 3-deoxy-d-*manno*-oct-2-ulosonic acid (Kdo); phosphoethanolamine (*P*Etn) and a one *O*-acetyl group (OAc). Additionally, both core oligosaccharides were de-*O*-acetylated and checked by ESI MS. The differences between core oligosaccharides and their de-*O*-acetylated fractions were related to the removal of the *O*-acetyl group from the structure. The major fraction represented by the ion at *m*/*z* 1159.30 [M+2H]^2+^ (Figure 2D) corresponded to the structure without an *O*-acetyl group in contrast with the ion at *m*/*z* 1180.42 [M+Ac+2H]^2+^ (Figure 2C). These two ions were selected for further analysis by use of positive ion mode ESI MS/MS. The main daughter ions detected in the ESI MS/MS spectra were explained. The ion at *m*/*z* 366.43 corresponds to the GalGlcNAc fragment, while the ion at *m*/*z* 407.48 was explained by the GlcNAcGalN-OAc (Figure 2C). The daughter ion with the highest *m*/*z* 569.59 was subsequently attributed to the GalGlcNAcGalN-OAc fragment. These observations, in comparison with NMR data, indicate that an *O*-acetyl group substitutes at Gal*p*N (residue **K’**).

The initial NMR investigation indicated the presence of hexosamine residue among the constituents of the core oligosaccharide; therefore, methylation analysis was performed on *N*-acetylated oligosaccharide. Methylation indicated the presence of 3,7-disubstituted Hep*p*, 3,4-disubstituted Hep*p*, 2-substituted Hep*p*, terminal Hep*p*, 4-substituted Glc*p*N, 4-substituted Gal*p*N, terminal Glc*p* and terminal Gal*p*.

**Figure 3:**
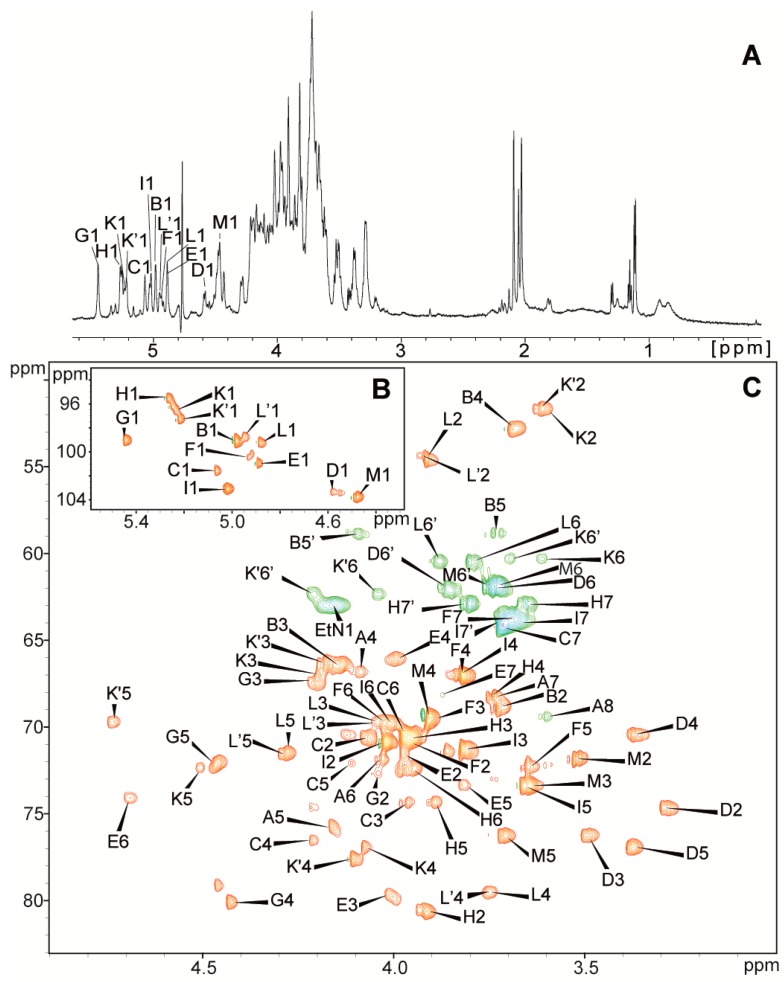
(**A**) The ^1^H NMR spectrum and (**B**,**C**) selected regions of the ^1^H-^13^C HSQC-DEPT spectrum of the core oligosaccharide *P. penneri* 40A LPS. The cross-peaks are labeled as shown in the text.

The ^1^H NMR spectrum of the *P. penneri* 40A core oligosaccharide (Figure 3A) contained the main signals for eleven anomeric protons, as well as signals characteristic for the deoxy protons from the Kdo residue. The ^1^H-^1^H COSY, TOCSY with different mixing times, ^1^H-^13^C HSQC-DEPT and HSQC-TOCSY spectra allowed for the assignments of the H-1 to H-6 (H-7,7′, H-8,8′) signals for each residue (marked as uppercase letters) of the core oligosaccharide (Table 2, Figure 3).

Residue **A** was recognized as a 5,8-disubstituted Kdo based on the characteristic deoxy proton signals of H-3ax (δ_H_ 1.80 ppm), H-3eq (δ_H_ 2.19 ppm) and a high chemical shift of the C-5 (δ_C_ 75.7 ppm) signal.

Residue **B** at δ_H_/δ_C_ 4.98/99.1 ppm, ^1^*J*_C-1,H-1_ ~ 170 Hz, was assigned as the terminal β-l-Ara4N residues based on the chemical shift of the C-4 (δ_C_ 52.8 ppm) and characteristic H5, H5′/C5 (δ_H_ 3.73, δ_H_/δ_C_ 4.10/58.8 ppm) signals.

Residue **C** at δ_H_/δ_C_ 5.07/101.6 ppm, ^1^*J*_C-1,H-1_ ~ 170 Hz, was recognized as a 3,4-disubstituted l-*glycero*-α-d-*manno*-Hep*p* based on the relatively high chemical shift of the C-3 (δ_C_ 74.4 ppm) and C-4 (δ_C_ 76.5 ppm) signals.

Residue **D** at δ_H_/δ_C_ 4.58/103.3 ppm, ^1^*J*_C-1,H-1_ ~ 162 Hz, was assigned as the terminal β-d-Glc*p* based on the large vicinal couplings between all protons in the sugar ring.

Residue **E** at δ_H_/δ_C_ 4.89/101.0 ppm, ^1^*J*_C-1,H-1_ ~ 172 Hz, was recognized as the 3,7-disubstituted l-*glycero*-α-d-*manno*-Hep*p* from the relatively high chemical shifts of the C-3 (δ_C_ 79.7 ppm) and C-7 (δ_C_ 68.2 ppm) signals.

Residue **F** at δ_H_/δ_C_ 4.93/100.4 ppm, ^1^*J*_C-1,H-1_ ~ 168 Hz, as well as residue **I** at δ_H_/δ_C_ 5.02/103.1 ppm, ^1^*J*_C-1,H-1_ ~ 170 Hz, were assigned as terminal l-*glycero*-α-d-*manno*-Hep*p.*

Residue **G** at δ_H_/δ_C_ 5.45/99.0 ppm, ^1^*J*_C-1,H-1_ ~ 168 Hz, was assigned as the 2,4-disubstituted α-d-Gal*p*A residues based on the characteristic five proton spin systems, and the high ^13^C chemical shift of the C-2 (δ_C_ 72.7 ppm), C-4 (δ_C_ 80.1 ppm) and C-6 (δ_C_ 176.0 ppm) signals.

Residue **H** at δ_H_/δ_C_ 5.27/95.5 ppm, ^1^*J*_C-1,H-1_ ~ 173 Hz, was recognized as a 2-substituted d-*glycero*-α-d-*manno*-Hep*p* based on the relatively high chemical shift of the C-2 (δ_C_ 80.6 ppm) signal.

Additionally, the characteristic chemical shift of the C-6 (δ_C_ 72.4 ppm) signal indicates the d-*glycero*-d-*manno* configuration [11].

Residue **K’** at δ_H_/δ_C_ 5.22/97.3 ppm, ^1^*J*_C-1,H-1_ ~175 Hz, was recognized as the 4-substituted α-d-Gal*p*N6OAc based on the chemical shift of the C-2 (δ_C_ 51.7 ppm) signal, the relatively high chemical shift C-4 (δ_C_ 77.6 ppm) signal, and the downfield shift of the C-6 (δ_C_ 62.3 ppm) signal, consistent with an *O*-acetyl group at position 6. The presence of an *O*-acetyl group was supported by de-*O*-acetylation. The spectrum of the de-*O*-acetylated core oligosaccharide contained only one signal from the *N*-acetyl group of residue **L** whereas OS also possesses an additional signal from the *O*-acetyl group at Gal*p*N (residue **K’**). Residue **K** at δ_H_/δ_C_ 5.23/96.5 ppm, ^1^*J*_C-1,H-1_ ~ 174 Hz, represented a variant of residue **K’** caused by the lack of the *O*-acetyl group at *O*-6 of residue **K’**. Residue **K** was thus identified as 4-substituted α-d-Gal*p*N.

Residue **L** at δ_H_/δ_C_ 4.88/99.2 ppm, ^1^*J*_C-1,H-1_ ~ 177 Hz, was recognized as the 4-substituted α-d-Glc*p*NAc based on the chemical shift of the C-2 (δ_C_ 54.6 ppm) signal, the relatively high chemical shift of the C-4 (δ_C_ 79.5 ppm) signal. Residues **L’** at δ_H_/δ_C_ 5.40/102.4 ppm, ^1^*J*_C-1,H-1_ ~175 Hz, were recognized as variants of residue **L** (4-substituted α-d-Glc*p*NAc) due to the absence of the *O*-acetyl group located at position 6 of residue **K’**.

Residue **M** at δ_H_/δ_C_ 4.48/103.7 ppm, ^1^*J*_C-1,H-1_ ~ 162 Hz, was recognized as the terminal β-d-Gal*p* based on chemical shifts in good agreement with those of previously reported β-d-Gal*p* [12].

**Figure 4:**
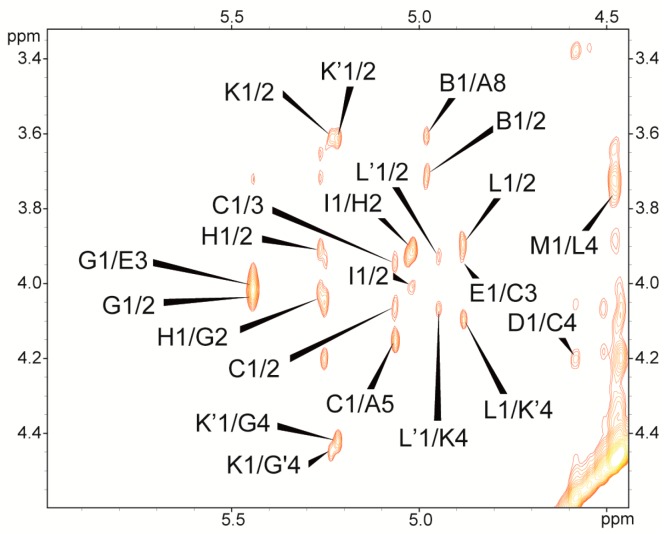
Selected part of the NOESY spectrum of the core oligosaccharide of *P. penneri* 40A LPS. The cross-peaks are labeled as shown in the text.

Each disaccharide element of the core oligosaccharide was identified by ^1^H-^1^H NOESY (Figure 4) and ^1^H-^13^C HMBC experiments. The NOESY spectrum showed strong inter-residue cross-peaks between the transglycosidic protons: H-1 of **B**/H-8 of **A**, H-1 of **C**/H-5 of **A**, H-1 of **D**/H-4 of **C**, H-1 of **E**/H-3 of **C**, H-1 of **G**/H-3 of **E**, H-1 of **H**/H-2 of **G**, H-1 of **I**/H-2 of **H**, H-1 of **K**/H-4 of **G**, H-1 of **L**/H-4 of **K’**, H-1 of **L’**/H-4 of **K** and H-1 of **M**/H-4 of **L** (Figure 4). These data confirmed the substitution positions of the monosaccharide residues and demonstrated their sequence in the core oligosaccharide *P. penneri* 40A (structure inserted into Figure 2).

## 3. Discussion

This work provides the serological and chemical characterization of a new type of the core region presented by *P. penneri* 40A and 41 LPSs. In ELISA, anti-conjugate serum *P. penneri* 40A reacted differently with three groups: (I) *P. penneri* 40A and 41—showing the strongest serological activity; (II) *P. penneri* 4 and 1—weaker reactions; and (III) *P. penneri* 27 and 71—the weakest serological activity. The weakest activity of the last two LPSs was also confirmed by the results of the Western blot technique (Figure 1). The LPS whose binding-pattern of low-molecular-mass LPS species distinguished itself from the patterns of the tested residual LPSs was *P. penneri* 1. The LPS, *P. penneri* 4, reacted in Western blotting similarly to *P. penneri* 40A and 41 but its reactivity titer in ELISA was twice as low as the titers of *P. penneri* 40A and 41 LPSs. These differences in serological activity of *P. penneri* 1, 4, 27 and 71 LPS core oligosaccharides compared to that observed for *P. penneri* 40A and 41 LPSs suggest that these two groups of LPSs share common epitopes but do not necessarily present the same sero- and chemotype of the core region. In many cases, LPSs, presenting one chemotype of the core region, showed similar binding-patterns of low-molecular-mass LPS species in the Western blot technique and reacted in ELISA up to the same value of the antiserum reactivity titers [7,8]. Due to the fact that *P. penneri* 40A and 41 LPSs reacted similarly in all serological assays, they were chosen for the structural analyses.

The results of mass spectra analyses of the core oligosaccharides from *P. penneri* 40A and *P. penneri* 41 LPS were able to reveal the structure, which had not been previously identified for *Proteus* LPS core regions. The new type of structure is typical for *P. penneri* core regions in its inner part containing five Hep*p* residues, Glc*p*, Gal*p*A, Kdo and Ara4N residues and presenting III glycoform [2,4]. Only in six *P. penneri* LPSs 12, 13, 14, 37, 42 and 44, can structural variations of the inner core region be observed [2,4]. The uniqueness of *P. penneri* 40A and 41 core regions is found in its outer part defined in the literature as R substituent [4]. To date, 20 different structures of R substituent have been determined for *Proteus* spp. strains, among which 12 are presented by *P. penneri* strains [4,13]. These structures contain from one (e.g., *P. penneri* 12, 13) to four sugar residues or their *N*-acetylated forms (e.g., *P. penneri* 7, 14, 15). One structure of R substituent can be represented by one or a few *P. penneri* strains [4]. The R substituent of *P. penneri* 40A (O64a,b,d) and the 41 (O62) LPS core region is built of three residues (β-d-Gal*p*-(1→4)-α-d-Glc*p*NAc-(1→4)-α-d-Gal*p*N6OAc) and it is similar to the outer core region of only one strain, *P. penneri* 103 (O73a,b) (β-d-Glc*p*-(1→4)-α-d-Glc*p*NAc-(1→4)-α-d-Gal*p*N6OAc) [4]. These two fragments differ from each other in the terminal residue. The importance of the terminal residue in the serospecificity of the outer core oligosaccharide region can be confirmed by the fact that *P. penneri* 40A anti-conjugate serum did not react with *P. penneri* 103 LPS. *P. penneri* 103 was classified into serotype group no. 10 together with *P. penneri* 75 LPS, recognized by anti-core-specific antibodies present in *P. penneri* 103 antiserum [10]. A similar situation also occurs in the case of two core oligosaccharide serotypes: R1 (α-d-Glc*p*-(1→4)-α-d-Gal*p*NAc-(1→2)-α-dd-Hep*p*-(1→6)-α-d-Glc*p*NGly) presented by *P. penneri* 7, 14 LPSs and R2 presented by *P. penneri* 8 LPS (α-d-Gal*p*NAc-(1→2)-α-dd-Hep*p*-(1→6)-α-d-Glc*p*NGly) differing in the lack of terminal residue [4,14]. The serological studies performed by use of *P. penneri* 7 core-specific antiserum and *P. penneri* 7, 8, 14 and 15 LPSs proved a crucial role of the terminal residue from the outer core region in its serospecificity [8]. As a result, *P. penneri* 8 LPS and *P. penneri* 7, 14 and 15 LPSs were classified into two different core oligosaccharide serotypes [10]. The 6-*O*-acetylation of the Gal*p*N residue is also unique for the *Proteus* spp. LPS core region. In *P. penneri* 16 and 18, Gal*p*N is substituted by the phosphoethanolamine group at position 6 [4].

The results presented in this work will allow *P. penneri* 40A and 41 LPSs to be classified into a new core oligosaccharide serotype group extending the core oligosaccharide serotypes scheme [10]. It is another example of two *P. penneri* LPSs of one core serotype but presenting two O serotypes: *P. penneri* 40A (O64a,b,d) and *P. penneri* 41 (O62)—the first representative of this O serogroup in the core types classification scheme.

Finding a new structure and serotype of the *P. penneri* LPS core region confirmed the huge structural heterogeneity of *P. penneri* LPSs, a unique phenomenon among other *Enterobacteriaceae*. Extension of the core serotype scheme with other representatives may be helpful in the identification of the most common R and O serotypes needed for the selection of vaccine antigens to obtain cross-reactive and cross-protective antibodies [10].

## 4. Materials and Methods

### 4.1. Bacterial Strains

*P. penneri* 40A (O64a,b,d) and 41 (O62) are clinical isolates from patients in Toronto (Canada) but their isolation sources remain unknown. The other strains, whose LPSs were checked with the tested serum, have been presented in another article [7]. All tested strains belong to the collection of the Laboratory of General Microbiology, University of Lodz (Poland), where they are stored in a glycerol mixture at −80 °C.

### 4.2. Lipopolysaccharide

The LPSs were extracted from dried bacterial cells, as previously described [7], by the phenol–water procedure according to the method of Westphal [15] and purified with aqueous 50% trichloroacetic acid. Alkali-treated LPSs used for the sera adsorption were prepared as described in detail elsewhere [16].

The LPSs of *P. penneri* 40A and 41 were degraded by treating with 1.5% acetic acid at 100 °C for 1 h and the carbohydrate portions were fractionated and monitored as described previously [7]. The fractions (*O*-PS, OS, and the mixture of low molecular mass) were eluted, freeze-dried and checked by ESI mass spectrometry and NMR spectroscopy.

### 4.3. De-O-Acetylation of the Core Oligosaccharide

The *P. penneri* 40A, 41 core oligosaccharides (5 mg) were treated with aqueous 12.5% NH_3_ (1 mL) at 23 °C for 16 h and then the solution was freeze-dried. The products were analyzed by ESI mass spectrometry and NMR spectroscopy.

### 4.4. P. penneri 40A Core Oligosaccharide Conjugate

Conjugation of the *P. penneri* 40A core oligosaccharide with diphtheria toxoid was performed by the method of H. J. Jennings and C. Lugowski based on the reaction of reductive amination, which was described in detail elsewhere [17]. The *P. penneri* 40A anti-conjugate serum was obtained by the immunization of New Zealand white rabbits as described previously [7].

### 4.5. Serological Assays

Purified LPS samples were tested with rabbit antisera in an enzyme-linked immunosorbent assay (ELISA), and Western blot procedure after sodium dodecyl sulfate polyacrylamide gel electrophoresis (SDS-PAGE) with non-adsorbed antisera and/or antisera adsorbed with selected alkali-treated LPSs. All assays were performed as previously described [16] with some modifications [7].

### 4.6. Chemical Method

Methylation analysis was performed according to the method of Ciucanu and Kerek [18]. Partially methylated alditol acetates were analyzed by gas chromatography-mass spectrometry using a Thermo Scientific ITQ system using a Zebron™ ZB-5HT (Thermo Fisher Scientific, Waltham, USA), GC Capillary Column (30 m × 0.25 mm × 0.25 μm) and with temperature rising from 150 to 270 °C at 8 °C/min.

### 4.7. Instrumental Methods

ESI MS analyses were performed using a Bruker microTOF-QII mass spectrometer (Bruker Brema, Germany) in a positive ion mode. The samples were dissolved in an acetonitrile–water–formic acid solution (50:50:0.1, *v*/*v*/*v*). The spectra were scanned in the *m*/*z* 200–2200 range. The mass isolation window for the precursor ion selection was set to 4 Da in the MS^2^ analyses.

All NMR spectra were recorded using a Bruker Avance III 600 Hz spectrometer equipped with a 2.5 mm microprobe, incorporating gradients along the z-axis. The measurements were performed at 298 K. The signals were assigned by one- and two-dimensional experiments: ^1^H-^1^H COSY, TOCSY (with mixing time: 30, 60, 100 ms), NOESY and ^1^H-^13^C HSQC-DEPT, HSQC-TOCSY, and HMBC. The data were acquired and processed using standard Bruker software. The processed spectra were assigned with the help of the SPARKY program [19].

## Figures and Tables

**Table 1:** ESI MS data obtained for the core oligosaccharides of *P. penneri* 40A and 41.

The Observed Ion (*m*/*z*)/The Calculated Mass		The Ion Interpretation
*P. penneri* 40A	*P. penneri* 41	
1180.42^2+^/2358.84	1180.41^2+^/2358.82	[M+Ac+2H]^2+^
1171.40^2+^/2340.80	1171.41^2+^/2340.82	[M+Ac-H_2_O+2H]^2+^
1159.30^2+^/2316.60	1159.41^2+^/2316.82	[M+2H]^2+^
1150.41^2+^/2298.82	1150.40^2+^/2298.80	[M-H_2_O+2H]^2+^
1114.90^2+^/2227.80	1114.87^2+^/2227.74	[M-Ara4N+Ac+2H]^2+^
1106.40^2+^/2210.80	1106.37^2+^/2210.74	[M-Ara4N+Ac-H_2_O+2H]^2+^
1099.70^2+^/2197.40	1099.87^2+^/2197.74	[M-Hex+Ac+2H]^2+^
1090.90^2+^/2179.80	1090.86^2+^/2179.72	[M-Hex+Ac-H_2_O+2H]^2+^
1084.90^2+^/2167.80	1084.86^2+^/2167.72	[M-Hep+Ac+2H]^2+^
1078.90^2+^/2155.80	1078.86^2+^/2155.72	[M-Hex+2H]^2+^

**Table 2:** ^1^H and ^13^C NMR chemical shifts of the core oligosaccharide of *P. penneri* 40A LPS.

Residues	Chemical Shifts (ppm)							
	H1/C1	H2/C2	H3(H3ax,eq)/C3	H4/C4	H5,5′/C5	H6,6′/C6	H7,7′/C7	H8,8′/C8 (NAc, OAc)
A→5,8)-Kdo			(1.80, 2.19)	4.09	4.16	4.03	3.74	3.60, 3.92
			35.4	66.8	75.7	71.7	68.2	69.4
B β-l-Ara4N-(1→	4.98	3.72	4.14	3.68	3.73, 4.10			
	99.1	68.8	66.5	52.8	58.8			
C→3,4)-l-*glycero*-α-d-*manno*-Hep*p*-(1→	5.07	4.06	3.96	4.21	4.11	3.97	3.72	
	101.6	70.6	74.4	76.5	72.1	70.5	64.3	
D β-d-Glc*p*-(1→	4.58	3.30	3.49	3.37	3.37	3.74, 3.86		
	103.3	74.7	76.3	70.4	76.9	62.0		
E→3,7)-l-*glycero*-α-d-*manno*-Hep*p*-(1→	4.89	3.98	4.01	3.99	3.81	4.69	3.88	
	101.0	71.6	79.7	66.1	73.3	74.1	68.2	
F l-*glycero*-α-d-*manno*-Hep*p*-(1→	4.93	3.96	3.89	3.82	3.65	4.02	3.69	
	100.4	70.9	69.5	67.1	72.4	69.8	63.8	
G→2,4)-α-d-Gal*p*A-(1→	5.45	4.04	4.21	4.43	4.46			
	99.0	72.7	67.3	80.1	72.2	176.0		
H→2)-d-*glycero*-α-d-*manno*-Hep*p*-(1→	5.27	3.91	4.00	3.75	3.89	3.95	3.65, 3.81	
	95.5	80.6	70.6	68.3	74.3	72.4	62.9	
I l-*glycero*-α-d-*manno*-Hep*p*-(1→	5.02	4.02	3.82	3.83	3.66	4.01	3.67, 3.72	
	103.1	70.8	71.3	67.0	73.4	69.8	64.0	
K→4)-α-d-Gal*p*N-(1→	5.23	3.60	4.19	4.08	4.50	3.61, 3.70		
	96.5	51.7	66.9	76.9	72.4	60.3		
K’→4)-α-d-Gal*p*N6OAc-(1→	5.22	3.62	4.18	4.11	4.73	4.04, 4.21		(2.10)
	97.3	51.7	66.3	77.6	69.7	62.3		(21.0, 174.5)
L→4)-α-d-Glc*p*NAc-(1→	4.88	3.92	4.04	3.75	4.28	3.79,3.88		(2.04)
	99.2	54.6	69.7	79.5	71.5	60.5		(22.7, 175.3)
L’→4)-α-d-Glc*p*NAc-(1→	4.95	3.93	4.05	3.78	71.5			(2.06)
	98.8	54.4	69.8	79.6	4.29			(22.7, 175.4)
M β-d-Gal*p*-(1→	4.48	3.53	3.64	3.91	3.71	3.72, 3.75		
	103.7	71.8	73.4	69.5	76.3	61.9		
PEtN	4.16	3.28						
	63.0	41.0						
